# Sleep alterations following COVID-19 are associated with both neuroinflammation and psychological disorders, although at different times

**DOI:** 10.3389/fneur.2022.929480

**Published:** 2022-08-17

**Authors:** Gaia Pellitteri, Andrea Surcinelli, Maria De Martino, Martina Fabris, Francesco Janes, Francesco Bax, Alessandro Marini, Romina Milanic, Antonella Piani, Miriam Isola, Gian Luigi Gigli, Mariarosaria Valente

**Affiliations:** ^1^Clinical Neurology Unit, Santa Maria della Misericordia University Hospital, Udine, Italy; ^2^Division of Medical Statistics, Department of Medical Area, University of Udine, Udine, Italy; ^3^Institute of Clinical Pathology, Santa Maria della Misericordia University Hospital, Udine, Italy; ^4^Neurology Unit, San Giovanni di Dio Hospital, Gorizia, Italy; ^5^Neurology Unit, Santa Maria della Misericordia University Hospital, Udine, Italy; ^6^Department of Medical Area (DAME), University of Udine, Udine, Italy

**Keywords:** SARS-CoV-2, sleep disorders, anxiety, psychological disorders, blood biomarkers, NfL, VCAM-1, COVID-19

## Abstract

**Introduction:**

By the end of 2019, severe acute respiratory syndrome coronavirus 2 rapidly spread all over the world impacting mental health and sleep habits. Insomnia, impaired sleep quality, and circadian rhythm alterations were all observed during the pandemic, especially among healthcare workers and in patients with acute and post-acute COVID-19. Sleep disruption may induce a pro-inflammatory state associated with an impairment of immune system function.

**Objective:**

We investigated the relationship between sleep alterations, psychological disorders, and inflammatory blood biomarkers in patients with post-acute COVID-19.

**Methods:**

We enrolled 47 subjects diagnosed with COVID-19 pneumonia at *Santa Maria della Misericordia* University Hospital (Udine, Italy) between March and May 2020. Selected patients were evaluated at 2 months (T1) and 10 months (T2) after discharge. Each time, we collected clinical interviews, neurological examinations, and self-administered questionnaires to assess sleep and life quality, anxiety, depression, and post-traumatic stress disorder. Blood biomarkers of endothelial activation, neuroinflammation, and inflammatory cytokines were also measured at each follow-up. Collected variables were analyzed using comparisons between groups and linear regression models.

**Results:**

Prevalence of insomnia increased from 10.6% up to 27.3% after COVID-19. Poor sleep quality was found in 41.5% of patients at both study visits. At T1 follow-up, poor sleepers showed higher levels of neurofilament light chain, vascular cell adhesion molecule 1, and interleukin 10; no significant associations were found between sleep quality and psychological disorders. At T2 follow-up, lower sleep quality was associated with higher levels of vascular cell adhesion molecule 1 and interleukin 8, but also with higher scores for anxiety, depression, and post-traumatic stress disorder.

**Conclusion:**

Our results suggest an association of poor sleep quality with both psychological disorders and neuroinflammation, although at different times, in previously hospitalized patients with moderate-to-critical COVID-19.

## Introduction

By the end of December 2019, a novel coronavirus was first identified in Wuhan (Hubei, China) and later defined as severe acute respiratory syndrome coronavirus 2 (SARS-CoV-2). The new virus, as the causative agent of coronavirus disease 2019 (COVID-19), rapidly spread all over the world and changed people's lives (1, 2). The ongoing COVID-19 pandemic has significantly impacted public health, the global economy, the labor market, education, social dynamics, and lifestyle (3). The fear of becoming infected, the disease itself, mandatory lockdowns, and quarantines have led to major consequences on mental health and sleep habits (4). A growing amount of literature is actually demonstrating how COVID-19 has severely affected sleep quality, sleep patterns, diagnosis, and management of sleep disorders (5). In a large meta-analysis on 44 studies and more than 54,000 participants from 13 different countries, the prevalence of sleep problems during the pandemic reached 32.3, 36, and 74.8% in the general population, healthcare workers, and COVID-19 patients, respectively (6). The term “COVID-somnia” has been suggested to embrace all sleep disorder symptoms commonly observed during the pandemic, both in response to psychological effects of social restrictions and COVID-19 acute and post-acute sequelae (5, 7).

A higher incidence of insomnia has been described particularly in women, young people, and patients with mood disorders and COVID-19 infection (8–12). Reduced sleep quality and alterations in sleep-wake circadian rhythm, such as delayed sleep phase, decreased nighttime sleep, and increased daytime napping, were observed during the pandemic; such disorders have been significantly associated with female sex, anxiety, depression, and higher stress levels (9, 13–16). An increased occurrence of nightmares related to COVID-19 was also observed, with similar risk factors (17–19). Beneficial effects on sleep, however, were found in adolescents due to decreased social jet lag and subsequent sleep deprivation (20–22). Healthcare workers have been profoundly affected by insomnia, reduced sleep quality, and nightmares, in association with mood disorders and chronic fatigue (23); frontline healthcare workers (i.e., medical staff directly working in COVID-19 care and management) have been found as the most severely involved (24, 25). Obstructive sleep apnea (OSA) shares with COVID-19 some elements of pathophysiology, including impaired ventilatory function and systemic inflammation. In addition, obesity constitutes an important common risk factor for both conditions. It is probably on this basis that patients with OSA were found with higher rates of hospitalization, intensive care admission, and mortality (26–29). Moreover, patients with OSA overexpress the angiotensin-converting enzyme 2 (ACE2) receptor in response to hypoxemia, which can explain the higher infection rate observed in this population (28, 30). Lastly, patients affected by COVID-19 showed increased sleep disorders both in acute and post-acute stages of the disease. Hospitalization has been recognized as a trigger factor for sleep impairment in those patients; a study has demonstrated sleep deterioration passing from 36% at the time of admission up to 69% in a week (31). Furthermore, a few studies described worse outcomes in hospitalized patients with COVID-19 classified as poor sleepers (32, 33). Sleep problems, especially insomnia, and reduced sleep quality are also common in the post-acute phase of the disease as part of the “long-COVID” syndrome (also called “long haulers” or “post-COVID”); the largest studies in this field showed a prevalence of about 25% of insomnia after six months from recovery (34–36).

The role of sleep disorders in compromising immune system function is well known (37, 38). Impaired sleep may have contributed to SARS-CoV-2 infection, weak response to COVID-19 vaccines, and poor outcomes in infected patients. Indeed, more frequent and prolonged hospitalizations and higher mortality rates have been found in COVID-19 patients with poor sleep quality, particularly in those with insomnia, daytime sleepiness and “long sleeper” phenotype (32, 39–41). Behind these observations, there is a pro-inflammatory state induced by sleep disruption, characterized by increased levels of norepinephrine, cortisol, and pro-inflammatory cytokines, decreased melatonin (and its anti-inflammatory, antioxidant, and immunomodulatory properties), reduced number and activity of natural killer (NK) cells of the innate immunity (32, 38, 42). On the other hand, a pro-inflammatory state itself may impair sleep, and inflammatory cytokines may alter sleep architecture (43, 44).

In the present study, we investigated the relationship between sleep alterations, psychological disorders, and blood biomarkers of inflammation in COVID-19. For this purpose, we followed for almost one year a sample group of patients who had been hospitalized during the first pandemic wave with a moderate-to-critical disease, according to the World Health Organization COVID-19 disease severity classification (45).

## Materials and methods

### Study design and patient selection

This retrospective and prospective observational study was performed at the Clinical Neurology Unit of *Santa Maria della Misericordia* University Hospital (Udine, Italy) between March 2020 and May 2021. This study was evaluated and approved by the Regional Ethics Committee of Friuli Venezia Giulia, Italy (Ref. No. ONCOV-2020-1) and was conducted in accordance with the Declaration of Helsinki code of ethics and the Good Clinical Practice guidelines. Written informed consent was obtained from all patients.

For the enrollment, we screened the patients consecutively admitted to COVID-19 general wards, semi-intensive and intensive care units of our hospital in the period between March 1^st^ and May 31^st^ 2020 (T0). All included patients were diagnosed with COVID-19 pneumonia based on a positive swab test and/or on clinical and radiological signs of lung involvement (Chest X-Ray or CT scan), requiring invasive and/or non-invasive ventilatory support. Patients who were unable to attend scheduled study visits or denied their informed consent were not included. Patients older than 75 years were also excluded in order to reduce the possible bias of pre-existing sleep alterations.

Selected patients underwent a clinical interview, a neurological examination, a comprehensive blood panel, and a broad spectrum of questionnaires at two different assessment times. The first visit was conducted 2 months after the patient's discharge (T1). Due to a new surge in the SARS-CoV-2 pandemic curve in the period between October 2020 and April 2021, the second visit (originally scheduled at 6 months) had to be delayed and was performed at a median time of 10 months (interquartile range, IQR 9.5-11) after discharge (T2).

### Data collection

The blood panel comprised a wide range of blood biomarkers, including mid-regional proadrenomedullin (MR-proADM), tumor necrosis factor-alpha (TNF-α), intercellular adhesion molecule 1 (ICAM-1), vascular cell adhesion molecule 1 (VCAM-1), neurofilament light chain (NfL), lipocalin 2 (NGAL), interleukin 1 beta (IL-1β), interleukin 2 receptor (IL-2R), interleukin 6 (IL-6), interleukin 8 (IL-8), interleukin 10 (IL-10), interferon-gamma (IFN-γ), and interferon gamma-induced protein 10 (IP-10/CXCL10). Mid-regional proadrenomedullin (MR-proADM) plasma concentrations were measured in an automated Kryptor analyzer, using TRACE technology (Kryptor, BRAHMS, Germany). All the other molecules were analyzed using a microfluidic ultrasensitive ELISA using the protein simple plex technology on the ELLA instrument (R&D systems, Biotechne, USA).

Self-administered questionnaires included the Pittsburgh Sleep Quality Index (PSQI) (46), Epworth Sleepiness Scale (ESS) (47), Beck Depression Inventory - Short Form (BDI-SF) (48), Hamilton Anxiety Rating Scale (HAS) (49), Impact of Event Scale-Revised (IES-R) (50), Short Form Health Survey 36 (SF-36) (51).

### Statistical analysis

Categorical variables were presented as an absolute value (percentage), continuous variables as mean ± standard deviation (SD) or median and IQR, as appropriate. Normality was assessed using the Shapiro–Wilk test. Categorical variables were compared using the chi-squared test or Fisher's exact test, while continuous variables were compared using a Student t-test or Mann–Whitney *U* test, according to the distribution of the data. Comparisons of dichotomous variables between the two follow-up times were performed using the McNemar test. Univariable and multivariable linear regression analyses were performed to estimate the association between PSQI scores at the two times of follow-up with demographic and clinical variables, by calculating the ß (linear regression coefficient) and 95% confidence intervals (CIs). Analyses were performed using Stata/IC 17.0 (StataCorp LP, College Station, USA).

## Results

Based on the enrollment criteria, 47 patients were included and underwent T1 evaluation. Three patients were lost to follow-up, while 44 attended the T2 study visits. At both times, however, blood tests could be obtained in 42 patients, while clinical questionnaires were completed by 41 subjects, as a matter of language barrier. Patients included were predominantly men (39, 83%), with a median age of 60 years (IQR 51-68; range 37–74). The more frequent comorbidities were arterial hypertension (20, 42.5%) and obesity (17, 36.2%), known as negative prognostic factors for COVID-19. Insomnia and anxiety were reported in the clinical history of five (10.6%) and four (8.5%) patients, respectively. During the acute phase of the disease, typical symptoms of COVID-19 were commonly observed, including fever, dyspnea, cough, myalgia, taste, and smell alterations. In addition, 12 patients (25.5%) experienced an acute confusional state while hospitalized. Invasive ventilatory support in the intensive care unit was required in 14 patients (29.8%), whereas the other 33 (70.2%) received non-invasive oxygen therapy in low and medium-intensity care units. The main demographics and clinical characteristics of patients at baseline and acute COVID-19 phase are summarized in Table 1.

**Table 1:** Main demographic and clinical characteristics of study sample at baseline.

**Demographics**	***N* = 47**
**Age (years)**, *median (IQR)*	60 (51–68)
**Female sex**, *n (%)*	8 (17.0)
**Main comorbidities** *, n (%)*	
Arterial hypertension	20 (42.5)
Obesity	17 (36.2)
Vascular diseases	8 (17.0)
Dyslipidemia	7 (14.9)
Diabetes	6 (12.8)
Cancer	2 (4.3)
Bronchial asthma	1 (2.1)
**Sleep disorders** *, n (%)*	
Insomnia	5 (10.6)
Obstructive sleep apnea	2 (4.3)
**Mood disorders**, *n (%)*	
Anxiety	4 (8.5)
**Acute COVID-19 clinical features**	
**Hospital ward**, *n (%)*	
Low/medium intensity care Intensive care unit Length of stay (days), *median (IQR)*	30 (63.8) 17 (36.2) 11 (7–21)
**Ventilatory support**, *n (%)*	
Venturi mask Non-invasive ventilation Orotracheal intubation OTI duration (days), *median (IQR)*	15 (31.9) 18 (38.3) 14 (29.8) 14.5 (10–18)
**COVID-19 acute symptoms**, *n (%)*	
Fever	46 (97.9)
Dyspnea	36 (76.6)
Cough	34 (72.3)
Myalgia	25 (53.2)
Taste disorders	25 (53.2)
Smell disorders	21 (44.7)
Headache	18 (38.3)
Diarrhea	14 (29.8)
Delirium	12 (25.5)
Syncope	4 (8.5)

N, number of patients; IQR, interquartile range; OTI, orotracheal intubation.

**Figure 1:**
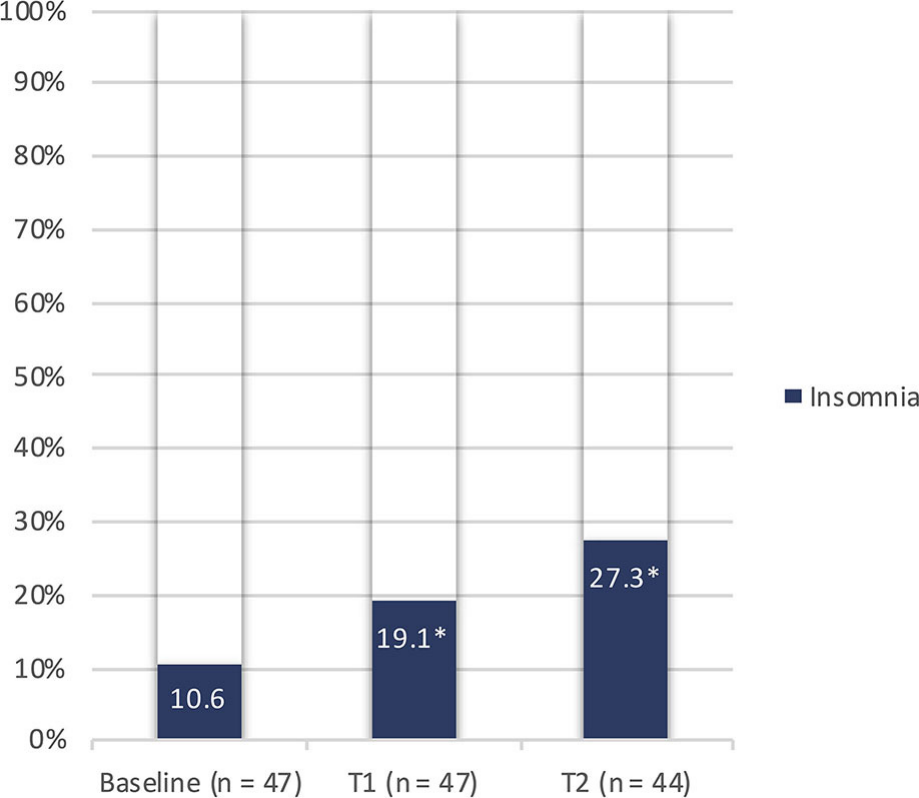
Prevalence of insomnia before and after COVID-19 in the study population. n, number; T1, 2-month study visit; T2, 10-month study visit; *p < 0.05 (0.045 and 0.02 by comparing baseline with T1 and T2, respectively).

At each study visit, a semi-structured interview was conducted in order to explore possible sleep disorders. Compared to basal conditions, at T1 and T2 we observed increasing rates of insomnia, defined in accordance with the international classification of sleep disorders, third edition (ICSD-3) (52). During the follow-up, prevalence of insomnia significantly increased from 10.6% (at baseline) to 19.1% (*p* = 0.045) and 27.3% (*p* = 0.02) at T1 and T2, respectively (Figure 1). Moreover, PSQI scores demonstrated a poor quality of sleep in 41.5% of patients 2 months after discharge; the same rate was confirmed at 10 months. Scores of single sub-items of PSQI did not significantly change between T1 and T2. Median PSQI total scores resulted higher in women at T1 (6 [IQR 4-9] *vs* 4 [IQR 3-7] in men, *p* = 0.421) and particularly at T2 (7.5 [IQR 6-9] vs. 5 [IQR 3-6] in men, *p* = 0.035). Despite the high percentage of poor sleepers, excessive daytime sleepiness according to the ESS score was found only in 4.9% and 7.3% of patients at T1 and T2, respectively (Table 2).

**Table 2:** PSQI and ESS total scores obtained at T1 and T2 study visits.

	**T1 (*N* = 41)**	**T2 (*N* = 41)**	***p***
**PSQI total score**, *mean ± SD*	5.4 ± 3.3	5.4 ± 2.8	0.536
Good sleepers (≤ 5), *n (%)*	24 (58.5%)	24 (58.5%)	
Poor sleepers (> 5), *n (%)*	17 (41.5%)	17 (41.5%)	
**ESS total score**, *mean ± SD*	4.3 ± 3.1	4.8 ± 3.2	0.633
Normal (≤ 10), *n (%)*	39 (95.1%)	38 (92.7%)	
Excessive (> 10), *n (%)*	2 (4.9%)	3 (7.3%)	

PSQI, Pittsburgh sleep quality index; ESS, Epworth sleepiness scale; n, number of patients; SD, standard deviation; T1, 2-month study visit; T2, 10-month study visit.

**Figure 2:**
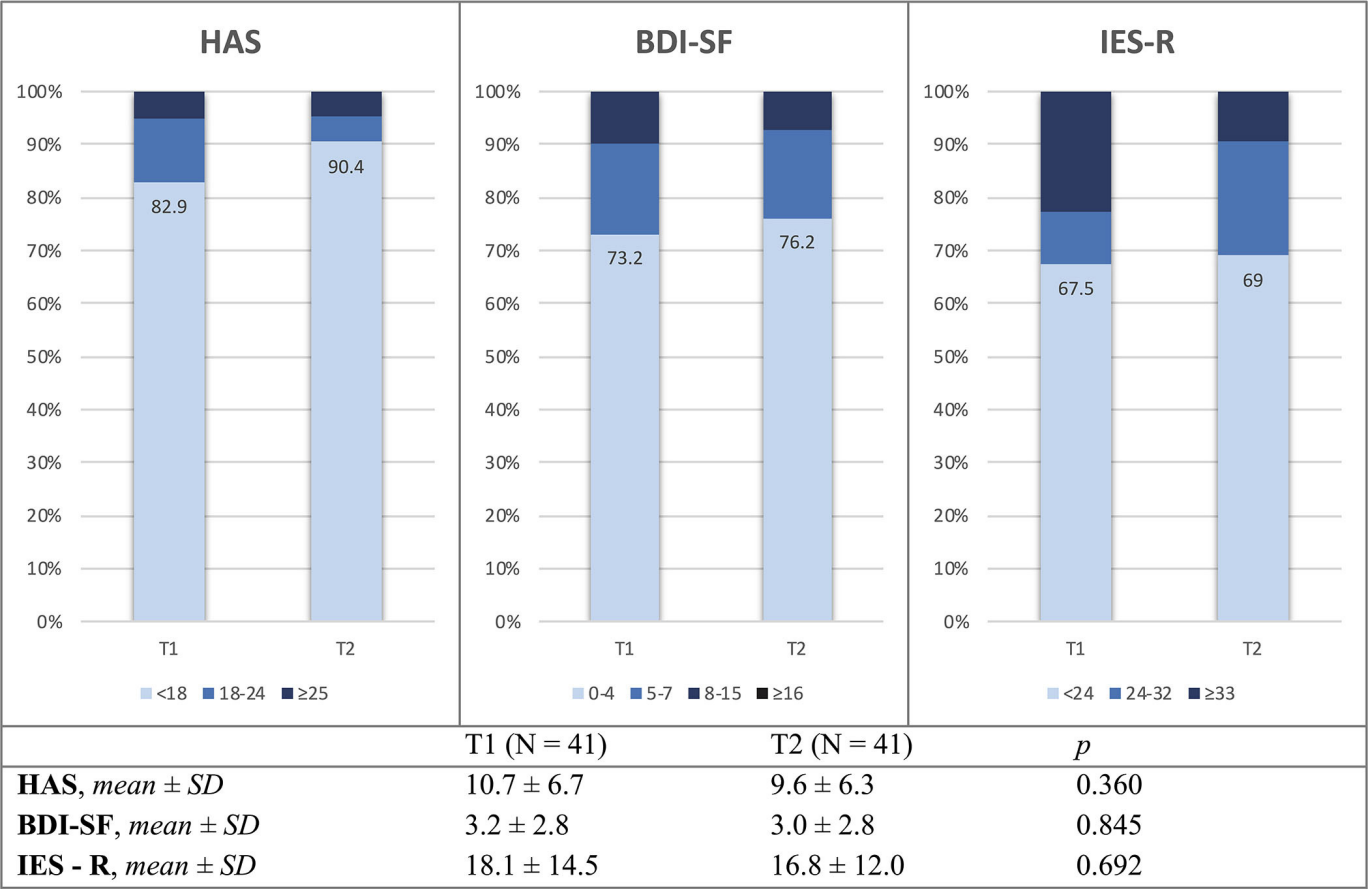
Mean values and distribution of HAS, BDI-SF, and IES-R scores at T1 and T2 study visits. HAS, Hamilton anxiety rating scale; BDI-SF, Beck depression inventory – short form; IES-R, impact of event scale – revised; n, number of patients; SD, standard deviation; T1, 2-month study visit; T2, 10-month study visit.

Clinical questionnaires evidenced a remarkable number of patients with abnormal T1 and T2 scores for anxiety (17.1 and 9.6%, respectively), depression (26.8 and 23.8%), and post-traumatic stress disorders (PTSD) (32.5 and 31%). However, this improving trend during the follow-up period did not reach statistical significance. Similarly, mean total scores for HAS, BDI-SF, and IES-R questionnaires did not significantly differ between T1 and T2 (Figure 2). A significant association was found between patients who had experienced delirium while hospitalized for COVID-19 and higher HAS scores at T2 visits; we found mean scores of 14.6 ± 7.4 in those with the previous delirium vs. 7.8 ± 4.8 in those without delirium (*p* = 0.001), indicating higher anxiety levels after COVID-19 in the first group.

Patients with abnormal PSQI total scores after COVID-19 showed worse quality of life at both evaluations. Several domains of the SF-36 questionnaire related to subjective physical and mental health were found significantly impaired in poor sleepers, compared to the good ones (Table 3). Considering the overall sample group, we observed a slight improvement in most of the SF-36 domains between T1 and T2, reaching the statistical significance only for the “physical role functioning” sub-item (median scores from 50 [IQR 25-100] up to 87.5 [IQR 25-100], *p* = 0.008).

**Table 3:** Relationship between poor sleep and life quality assessed with PSQI and SF-36 questionnaires at T1 and T2 study visits.

**SF-36 sections**	**PSQI ≤ 5** **(*N* = 24)** **Median (IQR)**	**PSQI > 5** **(*N* = 17)** **Median (IQR)**	***p***
**T1**			
Physical functioning	90 (80–95)	85 (65–95)	0.775
Physical role functioning	75 (25–100)	50 (25–75)	0.369
Bodily pain	84 (67–100)	52 (41–84)	0.045*
General health perceptions	76 (61–89)	67 (45–75)	0.023*
Vitality	70 (58–83)	55 (45–70)	0.011*
Social role functioning	81 (62–100)	62 (50–100)	0.189
Emotional role functioning	100 (66–100)	66 (33–100)	0.187
Mental health	88 (74–92)	76 (64–80)	0.008*
**T2**			
Physical functioning	95 (90–100)	85 (65–95)	0.030*
Physical role functioning	100 (75–100)	50 (25–100)	0.075
Bodily pain	92 (62.5–100)	52 (51–100)	0.073
General health perceptions	74 (64–91)	61 (45–76)	0.007*
Vitality	77.5 (60–85)	65 (50–70)	0.038*
Social role functioning	87 (75–100)	75 (62–100)	0.278
Emotional role functioning	100 (66–100)	66 (33–100)	0.094
Mental health	88 (80–96)	78 (72–84)	0.003*

SF-36 T1 and T2 scores were compared between subgroups of patients obtained using PSQI T1 and T2 scores, respectively. PSQI, Pittsburgh sleep quality index; SF-36, Short Form (36) Health Survey; IQR, interquartile range; N, number of patients; T1, 2-month study visit; T2, 10-month study visit;

^*^p < 0.05.

In order to evaluate the association between sleep quality and psychological distress in our sample, we performed two different univariable linear regression analyses using T1 and T2 PSQI total scores as the dependent variable, respectively. Both univariate analyses were adjusted for sex, as known influential factor on sleep quality and sleep disorders (53). T1 PSQI total score showed no significant association with HAS, BDI-SF, and IES-R T1 scores. Differently, using T2 PSQI total score, we found a significant association between higher T2 levels of anxiety, assessed by HAS questionnaire, and worse sleep quality (*p* = 0.004). However, no significant correlation between measures of depression and PTSD (BDI-SF and IES-R, respectively) was observed also at T2 visits. In both regression analyses, we did not find any association between PSQI total score and variables related to acute COVID-19 severity, including disease duration, length of stay, care intensity level, type, and duration of ventilatory support (Tables 4, 5).

**Table 4:** Univariate linear regression for PSQI total score at T1 study visit.

	**ß**	**95% CI**	***p***
**Baseline characteristics**			
Age	0.03	−0.07, 0.13	0.564
Sex	0.76	−2.04, 3.57	0.585
Ward *(ICU* vs. *other)*	−1.17	−3.32, 0.99	0.281
Microbiological recovery time	−0.09	−0.20, 0.02	0.096
Length of hospital stay	0.02	−0.04, 0.08	0.577
Ventilation type *(OTI* vs. *VM)*	−0.52	−3.27, 2.23	0.704
OTI duration	0.01	−0.01, 0.01	0.848
**T1 questionnaires**			
HAS	0.09	0.07, 0.25	0.249
BDI-SF	0.26	−0.11, 0.62	0.169
IES–R	−0.02	−0.09, 0.06	0.665
**T1 blood biomarkers**			
NfL	0.03	−0.04, 0.09	0.433
MR-proADM	4.36	−3.38, 12.10	0.261
ICAM-1	0.01	−0.01, 0.01	0.339
NGAL	0.01	−0.01, 0.02	0.644
VCAM-1	0.01	0.01, 0.01	0.001*
IL-1ß	−1.74	−6.25, 2.78	0.441
IL-6	0.24	−0.21, 0.70	0.285
IL-8	−0.01	−0.03, 0.03	0.811
TNFα	0.28	−0.11, 0.67	0.161
IL-2R	0.01	−0.01, 0.01	0.527
IL-10	0.72	0.04, 1.40	0.038*
IP 10	0.01	−0.02, 0.03	0.552
IFN-γ	1.17	−0.18, 2.52	0.088

All analyses have been adjusted for sex. PSQI, Pittsburgh sleep quality index; ß, linear regression coefficient; CI, confidence interval; ICU, intensive care unit; OTI, orotracheal intubation; VM, Venturi mask; HAS, Hamilton anxiety rating scale; BDI-SF, Beck depression inventory - short form; IES-R, impact of event scale – revised; NfL, neurofilament light chain; MR-proADM, mid-regional pro adrenomedullin; ICAM-1, intercellular adhesion molecule 1; NGAL, lipocalin 2; VCAM-1, vascular cell adhesion molecule 1; IL-1β, interleukin 1 beta; IL-6, interleukin 6; IL-8, interleukin 8; TNF-α, tumor necrosis factor alpha; IL-2R, interleukin 2 receptor; IL-10, interleukin 10; IP-10, interferon gamma-induced protein 10; IFN-γ, interferon gamma; T1, 2-month study visit; T2, 10-month study visit;

^*^p < 0.05.

**Table 5:** Univariate linear regression for PSQI total score at T2 study visit.

	**ß**	**95% CI**	***p***
**Baseline characteristics**			
Age	0.06	−0.02, 0.14	0.145
Sex	2.61	0.17, 5.05	0.037*
Ward *(ICU vs other)*	0.23	−1.61, 2.07	0.801
Microbiological recovery time	0.02	−0.08, 0.11	0.719
Length of hospital stay	0.04	−0.01, 0.09	0.114
Ventilation type *(OTI vs VM)*	0.70	−1.57, 2.97	0.537
OTI duration	0.03	−0.04, 0.10	0.360
**T1 questionnaires**			
HAS	0.04	−0.10, 0.17	0.612
BDI-SF	0.09	−0.22, 0.41	0.557
IES-R	0.01	−0.07, 0.07	0.972
**T2 questionnaires**			
HAS	0.19	0.06, 0.32	0.004*
BDI-SF	0.21	−0.09, 0.52	0.169
IES-R	0.06	−0.01, 0.13	0.102
**T1 blood biomarkers**			
NfL	0.02	−0.03, 0.08	0.414
MR-proADM	5.16	−0.96, 11.28	0.096
ICAM-1	−0.01	−0.01, 0.01	0.539
NGAL	0.01	−0.01, 0.02	0.899
VCAM-1	0.01	0.01, 0.01	0.034*
IL-1ß	−2.22	−5.89, 1.44	0.226
IL-6	0.22	−0.16, 0.59	0.248
IL-8	0.01	−0.02, 0.02	0.925
TNFα	0.28	−0.03, 0.60	0.077
IL-2R	0.01	−0.01, 0.01	0.202
IL-10	0.67	0.14, 1.20	0.014*
IP 10	0.01	−0.02, 0.02	0.691
IFN-γ	0.88	−0.26, 2.01	0.126
**T2 blood biomarkers**			
NfL	0.01	−0.08, 0.09	0.896
MR-proADM	4.17	−1.42, 9.76	0.139
ICAM-1	0.01	−0.01, 0.01	0.650
NGAL	0.01	−0.01, 0.03	0.126
VCAM-1	0.01	0.01, 0.01	0.044*
IL-1ß	−4.28	−45.44, 36.89	0.834
IL-6	−0.01	−0.56, 0.54	0.972
IL-8	0.20	0.02, 0.38	0.034*
TNFα	0.10	−0.23, 0.44	0.536
IL-2R	0.01	−0.01, 0.01	0.230
IL-10	0.32	−0.37, 1.01	0.354
IP 10	0.01	−0.01, 0.02	0.803
IFN-γ	0.43	−0.41, 1.26	0.305

All analyses have been adjusted for sex. PSQI, Pittsburgh sleep quality index; ß, linear regression coefficient; CI, confidence interval; ICU, intensive care unit; OTI, orotracheal intubation; VM, Venturi mask; HAS, Hamilton anxiety rating scale; BDI-SF, Beck depression inventory - short form; IES-R, impact of event scale – revised; NfL, neurofilament light chain; MR-proADM, mid-regional proadrenomedullin; ICAM-1, intercellular adhesion molecule 1; NGAL, lipocalin 2; VCAM-1, vascular cell adhesion molecule 1; IL-1β, interleukin 1 beta; IL-6, interleukin 6; IL-8, interleukin 8; TNF-α, tumor necrosis factor alpha; IL-2R, interleukin 2 receptor; IL-10, interleukin 10; IP-10, interferon gamma-induced protein 10; IFN-γ, interferon gamma; T1, 2-month study visit; T2, 10-month study visit;

^*^p < 0.05.

The same regression analyses, using T1 and T2 PSQI total scores, have been also extended to blood biomarkers in order to investigate the possible relationship between sleep disorders and an inflammatory or neuroinflammatory state. In univariate analyses, significant associations were observed between increased T1 levels of VCAM-1 (*p* = 0.001) and IL-10 (*p* = 0.038) with higher T1 PSQI total scores. Similarly, T2 PSQI total scores were found in a significant direct relationship with VCAM-1 (*p* = 0.034) and IL-10 (*p* = 0.014) T1 levels. In addition, T2 PSQI total scores were also associated with VCAM-1 (*p* = 0.044) and IL-8 (*p* = 0.034) T2 levels (Tables 4, 5).

As a further step, we tried to perform a multivariable regression analysis, but none of the previous results maintained statistical significance, probably due to the limited sample size.

**Figure 3:**
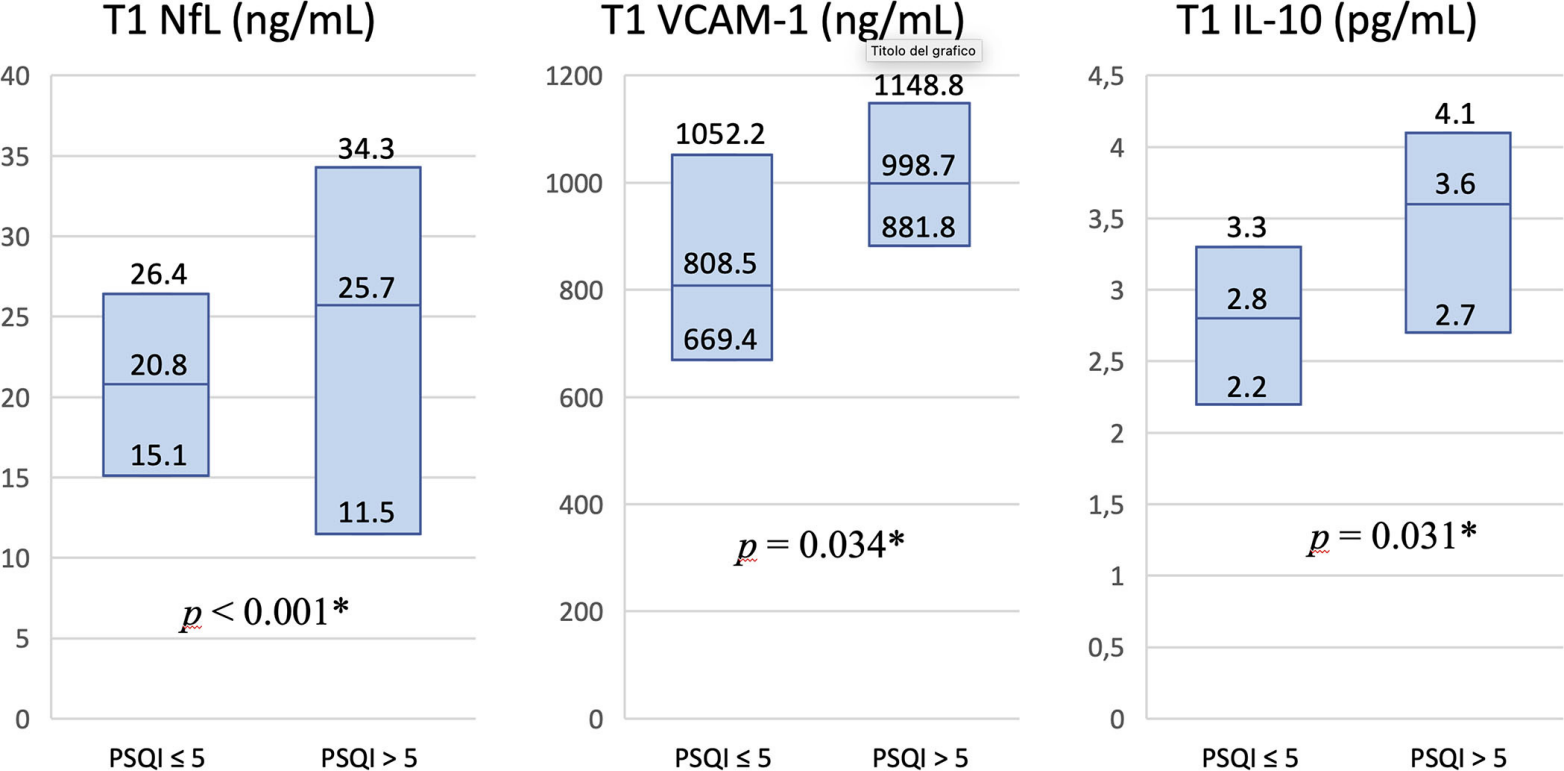
Differences in levels of blood biomarkers in patients with normal vs. abnormal PSQI total score at T1 study visit. Serum T1 levels of NfL, VCAM-1, and IL-10 were significantly higher in patients classified as poor sleepers at T1 evaluation. Values in columns are expressed as median and interquartile range. PSQI, Pittsburgh Sleep Quality Index; NfL, neurofilament light chain; VCAM-1, vascular cell adhesion molecule 1; IL-10, interleukin 10; T1, 2-month study visit; *p < 0.05.

We finally divided our sample into two subgroups, comparing blood biomarkers in patients with normal (≤ 5) versus abnormal (>5) PSQI total scores, both at T1 and T2. Poor sleepers at T1 showed significantly higher T1 levels of NfL (*p* < 0.001), VCAM-1 (*p* = 0.034), and IL-10 (*p* = 0.031) (Figure 3). On the contrary, none of the blood biomarkers was significantly different between poor and good sleepers at T2 evaluation.

The same subgroups were compared for questionnaires exploring psychological disorders. At T1 visits, HAS, BDI, and IES-R scores did not significantly differ between good and poor sleepers. At T2 visits, in contrast, HAS, BDI and IES-R scores were significantly higher in the subgroup of patients with PSQI > 5 compared to those with PSQI ≤ 5, with median HAS score of 12 (IQR 9-17) *vs* 7 (IQR 3-9), respectively (*p* < 0.001); median BDI-SF scores of 4 (IQR 2-6) *vs* 2 (IQR 0-2.5), respectively (*p* = 0.009); and median IES-R scores of 21 (IQR 17-25) *vs* 10.5 (IQR 5-17.5), respectively (*p* = 0.007) (Table 6).

**Table 6:** Median HAS, BDI-SF and IES-R scores in patients with normal (≤ 5) vs abnormal (> 5) PSQI total score at T1 and T2 study visits.

**T1**	**PSQI ≤ 5** **(*N* = 24)**	**PSQI > 5** **(*N* = 17)**	***p***
**HAS**, *median (IQR)*	8 (5–14)	11 (7–17)	0.199
**BDI-SF**, *median (IQR)*	2 (1–3.5)	4 (2–5)	0.077
**IES-R**, *median (IQR)*	12 (6–30)	19 (6–30)	0.658
**T2**			
**HAS**, *median (IQR)*	7 (3–9)	12 (9–17)	<0.001*
**BDI-SF**, *median (IQR)*	2 (0–2.5)	4 (2–6)	0.009*
**IES-R**, *median (IQR)*	10.5 (5–17.5)	21 (17–25)	0.007*

Subgroups were formed using T1 PSQI total scores for comparing HAS, BDI-SF and IES-R T1 scores, whereas T2 PSQI total scores were used for comparing HAS, BDI-SF and IES-R T2 scores. HAS, Hamilton anxiety rating scale; BDI-SF, Beck depression inventory - short form; IES-R, impact of event scale – revised; N, number of patients; IQR, interquartile range; T1, 2-month study visit; T2, 10-month study visit;

^*^p < 0.05.

## Discussion

In our study, we analyzed long-term sleep alterations in patients who had been previously hospitalized with moderate-to-critical COVID-19, requiring invasive and/or non-invasive ventilatory support. The patient's major complaint in the context of sleep disorders was an increased prevalence of insomnia, which passed from about 10% before COVID-19 up to 27% at a median interval of 10 months after the disease. Furthermore, the results of sleep questionnaires showed poor sleep quality in more than 40% of subjects, persisting both at 2 and 10 months after recovery. The higher rates of patients with poor sleep quality at PSQI scores, compared to self-reported sleep complaints at clinical interviews, may be explained by the greater sensitivity of questionnaires.

A first interesting finding is that long-lasting poor sleep quality in our patients did not appear to be related to COVID-19 severity during the acute phase. In order to investigate potential causes of insomnia and poor sleep quality, we explored possible psychological and biological concurrent factors in our sample. More specifically, we used HAS, BDI-SF, and IES-R questionnaires to evaluate levels of anxiety, depression, and PTSD. Overall, one-third of our patients showed abnormal scores on those scales, with a slight non-significant improvement during the follow-up. On the other hand, we had the opportunity to test all participants with a large number of blood biomarkers, including a wide cytokine panel (IL-1β, IL-2R, IL-6, IL-8, IL-10, TNF-α, IFN-γ, IP-10), endothelial adhesion molecules (ICAM-1 and VCAM-1), markers of organ damage (MR-proADM, NGAL), neutrophil and microglial activation (NGAL), and neuronal injury (NfL).

At 2 months after the acute phase of COVID-19, we found that patients with lower sleep quality had higher levels of NfL, VCAM-1, and IL-10, but no significant associations were found with HAS, BDI-SF, and IES-R scores. At 10 months after acute COVID-19, poor sleepers similarly showed higher levels of VCAM-1 and IL-10 on T1 blood samples, but higher VCAM-1 and IL-8 levels on T2 blood samples. At T2 follow-up, differently from T1, poor sleepers showed greater levels of anxiety, depression, and PTSD on the respective questionnaires. Based on these clinical-laboratory findings, we can hypothesize that poor sleep quality at T1 follow-up was mostly associated with a neuroinflammatory condition and neuronal damage (especially considering NfL serum levels), but not with a specific psychological profile. On the contrary, while inflammatory biomarkers progressively faded in the following months, a significant correlation between worse sleep quality and psychological disorders, particularly anxiety, emerged at T2 follow-up (these two main clinical-laboratory patterns have been summarized in Table 7). In this regard, we incidentally observed that more anxious subjects at the second follow-up were those who had experienced delirium while hospitalized. In other studies, both delirium and anxiety have been found to increase in patients with COVID-19 (54, 55). Actually, dysexecutive symptoms including confusion and behavioral disorders had already been observed in other coronaviruses infections and in acute respiratory distress syndromes in general (56, 57). Delirium and anxiety, therefore, could be both related to a dysexecutive pattern associated with COVID-19 (58, 59).

**Table 7:** Summary of main clinical-laboratory patterns in poor sleepers at T1 and T2 study visits.

	**T1 poor sleepers**	**T2 poor sleepers**
NfL	✓	
IL-10	✓	
VCAM-1	✓	✓
IL-8		✓
Anxiety		✓
Depression		✓
PTSD		✓

NfL, neurofilament light chain; IL-10, interleukin 10; VCAM-1, vascular cell adhesion molecule 1; IL-8, interleukin 8; PTSD, post-traumatic stress disorder; T1, 2-month study visit; T2, 10-month study visit.

A few studies in the literature have documented prolonged follow-up periods in patients with a history of COVID-19. Groff et al. conducted a systematic review on short- and long-term sequelae of SARS-CoV-2 infection, finding only nine studies with a minimum follow-up of six months. Overall, one in four COVID-19 survivors was diagnosed with sleep disorders, one in three with generalized anxiety disorder, one in five with depression, and one in eight with PTSD (36). Our results are quite consistent with the literature, except for a slightly higher prevalence of psychological disorders. This could be explained by the fact that only patients with moderate-to-critical COVID-19, with a possible higher impact on mental health, were enrolled in our study.

Our results suggest an association between poor sleep quality and both neuroinflammation and psychological disorders, although at different times. However, our data are insufficient to draw conclusions about any causal relationship between these factors. Nevertheless, it is reasonable to speculate on a mutual influence among these conditions. Other studies have already evaluated the association between sleep disorders and mood alterations in patients with COVID-19. Bacaro et al. found insomnia severity after SARS-CoV-2 infection significantly associated with both anxiety and depression (8), whereas Akinci and Başar with depression only (32). Indeed, the reciprocal relationship between anxiety and depression with insomnia was already known long before than COVID-19 pandemic (60, 61).

Sleep disruption and neuroinflammation are likely to influence each other as well. Insomnia and sleep deprivation are known to be associated with a low-grade pro-inflammatory state, characterized by the release of acute-phase proteins and cytokines, including IL-1, IL-6, and TNF-α (32, 62). In particular, IL-1 and TNF-α contribute to non-rapid eye movement (NREM) sleep regulation under physiological and inflammatory conditions; at lower dosages, these molecules increase NREM sleep. With increasing dosages, they first inhibit rapid eye movement (REM) sleep, and then both REM and NREM sleep (43). On the contrary, IL-10 and other anti-inflammatory cytokines are known to inhibit IL-1 and TNF-α, thus attenuating NREM sleep (43, 44).

In our sample, we found no relationship between sleep quality and blood levels of IL-1, IL-6, and TNF-α at any time of the follow-up. Unfortunately, cytokine dosages at the T0 acute phase of the disease are not available, but it would be interesting to find out whether short- and long-term poor sleepers were those with higher pro-inflammatory cytokines (particularly IL-1, IL-6, and TNF-α) during the hospitalization. Nonetheless, we found an increased expression of VCAM-1 in patients with poor sleep quality at both times of follow-up. This biomarker is expressed by the endothelial cells in case of inflammation, facilitating leukocytes adhesion to vascular endothelium. IL-8, which was also found to increase in patients with poor sleep quality at T2 follow-up, is similarly expressed by endothelial cells and it contributes to leukocytes chemotaxis, primarily neutrophils (63). What is remarkable is that VCAM-1 and IL-8 upregulation are both mediated by IL-1 and TNF-α (63–65). So, on a speculative plan, it is possible that the post-acute overexpression of VCAM-1 and IL-8 in poor sleepers could be somehow influenced by higher levels of pro-inflammatory cytokines (including IL-1 and TNF-α) during the acute phase of COVID-19. In order to add information on the regulation mechanisms between those cytokines, it would be useful to measure serum levels of IL-1 and TNF-α during the acute phase of COVID-19, testing their correlation with acute and post-acute VCAM-1 and IL-8 values. The meaning of the increased levels of IL-10 in patients with poor sleep quality at 2 months of follow-up remains unclear, but we might hypothesize a sort of compensatory response of the immune system at the end of the acute phase of COVID-19, with increased levels of anti-inflammatory cytokines inhibiting the release of pro-inflammatory mediators.

Moreover, there is emerging evidence on the role of sleep disorders in inducing neuroinflammation. Semyachkina-Glushkovskaya et al. have recently published a thorough review focused on the possible relationship between sleep loss, neuroinflammation, and disruption of the blood-brain barrier (BBB) in COVID-19 patients (62). As reported above, systemic inflammation and pro-inflammatory cytokines lead to changes in endothelial cells, also involving cerebral vessels with potential alterations in brain permeability. This review actually summarized a large number of studies on this topic, performed before the COVID-19 pandemic, which had demonstrated an increased BBB permeability to inflammatory mediators in response to sleep deprivation. In our post-acute COVID-19 patients, we found higher T1 levels of serum NfL in poor sleepers; this can be interpreted as a sign of neuroinflammation and neuronal damage associated with sleep disruption. However, NfL levels tended to normalize over time, and the association with poor sleep quality was not confirmed at T2 follow-up. Similar findings have been reported in two other recent studies on the long-COVID syndrome, in which NfL serum levels decreased after 6 months of follow-up and did not significantly differ between patients who had long-COVID symptoms and those who did not (66, 67).

## Study limitations and strengths

Our study has several limitations. First of all, the patients were actively tested only in the post-acute phase of COVID-19, whereas baseline information and acute disease data were collected retrospectively. In particular, clinical questionnaires and blood panels were not performed before or during the acute phase of COVID-19; this could have limited a proper interpretation of those variables and their changes over time. Secondly, the limited sample size could have contributed to the weakness of results obtained in multivariable regression analyses. Finally, we evaluated insomnia and quality of sleep based on clinical interviews and PSQI questionnaires, respectively, rather than using objective measures such as polysomnography.

On the other hand, our study also has several strengths. The first one is the long-term follow-up, regarding both self-administered questionnaires and blood biomarkers. Moreover, our blood panel included a broad spectrum of biomarkers, reflecting neuroinflammation, endothelial vascular activation, and changes in immune system regulation.

## Conclusion

After COVID-19, the prevalence of insomnia increased by 17% and poor sleep quality was found in more than 40% of patients after 10 months of follow-up. Our study suggests that neuroinflammation first and then psychological disorders, particularly anxiety, play a role in COVID-19-related sleep disruption. Mood and sleep alterations could be also interpreted as part of the neuro-homeostatic response to transient neuronal damage due to SARS-CoV-2 induced pro-inflammatory state. All these conditions seem to influence each other in a complex relationship that remains partially unclear.

Longer follow-up studies on larger groups of patients are needed to evaluate the dynamics of sleep disorders and other long-COVID symptoms over time. In this regard, blood biomarkers are gaining even more importance as prognostic factors and useful tools in understanding disease pathophysiology. From a clinical point of view, we recommend screening of patients with acute and post-acute COVID-19 for sleep disorders. Improving sleep quality with appropriate treatments can help in reducing neuroinflammation, disease severity, and hospital admission, also increasing immune system response to infections and vaccines, and, more generally, the life quality of patients.

## Data availability statement

The raw data supporting the conclusions of this article will be made available by the authors, without undue reservation.

## Ethics statement

The studies involving human participants were reviewed and approved by Comitato Etico Unico Regionale del Friuli Venezia Giulia (CEUR FVG). The patients/participants provided their written informed consent to participate in this study.

## Author contributions

GP, AS, FB, AM, MI, MV, and GG participated in the conception and design of the study. GP, AS, FB, AM, FJ, AP, MF, and RM acquired the data. GP, MF, and MD analyzed the data. GP, AS, GG, MF, and MD drafted the manuscript for intellectual content. GP, FJ, GG, and MV revised the manuscript for intellectual content. All authors contributed to the article and approved the submitted version.

## Funding

This study was supported in part by available funds at *Santa Maria della Misericordia* University Hospital (Udine, Italy) raised through public and private donations for the COVID-19 emergency.

## Conflict of interest

The authors declare that the research was conducted in the absence of any commercial or financial relationships that could be construed as a potential conflict of interest.

## Publisher's note

All claims expressed in this article are solely those of the authors and do not necessarily represent those of their affiliated organizations, or those of the publisher, the editors and the reviewers. Any product that may be evaluated in this article, or claim that may be made by its manufacturer, is not guaranteed or endorsed by the publisher.
